# Effective coverage of antenatal care service utilisation in Ethiopia using service provision assessment and demography and health survey data

**DOI:** 10.1177/17455057261438733

**Published:** 2026-07-24

**Authors:** Ayal Debie, Molla M. Wassie, Claire T. Roberts, Annabelle Wilson, Jacqueline H. Stephens

**Affiliations:** 1 Flinders Health and Medical Research Institute, College of Medicine and Public Health, Flinders University, Adelaide, SA, Australia; 2 Department of Health Systems and Policy, Institute of Public Health, University of Gondar, Gondar, Ethiopia

**Keywords:** antenatal care, Ethiopia, effective coverage, service provision assessment

## Abstract

**Background:**

Globally, maternal deaths due to pregnancy and childbirth-related causes are still high. In response to the high maternal deaths, countries committed to achieving universal health coverage (UHC) by 2030. Effective coverage (EC) is a key metric for tracking the progress in the service coverage component of UHC.

**Objectives:**

This study aimed to assess the magnitude of EC of four or more antenatal care (ANC4+) in Ethiopia.

**Design:**

Ethiopian Mini-Demography and Health Survey (EMDHS) 2019 and Ethiopian Service Provision Assessment (ESPA) 2021–2022 cross-sectional surveys were used for analysis.

**Methods:**

EC of ANC4+ was estimated using the EMDHS 2019 with a weighted sample of 3617 women with their most recent live birth and ESPA 2021–2022 data with a sample of 659 health facilities. Accordingly, crude coverage (CC) was calculated from the EMDHS 2019, while a quality score was calculated from the ESPA 2021–2022 data. ArcGIS Pro software was used to display EC of ANC4+ across the administrative regions of Ethiopia.

**Results:**

In this study, the overall CC and EC of ANC4+ were 41.5% (95% CI: 38.9, 44.0) and 21.6% (95% CI: 20.0, 23.2), respectively with the lowest in Somali region and the highest in Addis Ababa. The overall facility-weighted quality of ANC was 52.1% (95% CI: 51.3, 52.8) with the lowest in Gambela and the highest in Benishangul-Gumuz region.

**Conclusion:**

This study revealed that the EC of ANC4+ was low due to low achievement in the CC of ANC4+ and quality of care with high regional disparities. Without urgent action, Ethiopia is unlikely to meet Sustainable Development Goal-3 (SDG-3) targets for maternal health. Therefore, administrative region-specific intervention strategies should be designed to improve community engagement, infrastructure, access, and quality of ANC in Ethiopia.

## Introduction

About 287,000 women die each year (about 800 women die every day) of pregnancy and childbirth-related causes worldwide in 2020.^1^ This is despite the maternal mortality ratio (MMR) having dropped by 34% worldwide between 2000 and 2020.^1^ Maternal deaths in Sub-Saharan Africa (SSA) account for over two-thirds of the global burden and MMR is projected to remain high, with an estimated 390 deaths per 100,000 live births by 2030.^1,2^ In SSA region, haemorrhage (28.8%), hypertensive disorders (22.1%), non-obstetric complications (18.8%), and infections (11.5%) were identified as the commonest causes of maternal deaths.^3^ Ethiopia also reduced its maternal deaths by 70% between 1990 and 2013.^4^ Despite this progress, the MMR remained high at 412 deaths per 100,000 live births in 2016^5^ and 141 in 2024–2025.^6^

The United Nations Member States committed to achieving the 2030 Sustainable Development Goals (SDGs) including universal health coverage (UHC).^7^ SDG-3 specifically focuses on ensuring healthy lives and promoting well-being for all at all ages.^8^ UHC is central to SDG-3 which is an approach to providing quality and equitable essential health service to all without any financial hardship.^7^ Effective coverage (EC) is the preferred indicator for monitoring the service coverage dimension of UHC and captures a nation’s efforts to meet people’s needs.^8^ EC unites service utilisation and quality of care into a single metric to measure health system performance and the potential health gain delivered to the population.^9^ EC of four or more antenatal care (ANC4+) service utilisations is the key indicator to measure the progress of the service coverage aspects of maternal healthcare service towards UHC.

A study in Cambodia indicated crude coverage (CC), and EC were 80.1% and 56.4% for maternal health services, respectively.^10^ In Pakistan, only 35% of pregnant women received EC of ANC among 92% of ANC4+ users.^11^ Ethiopia is a nation with low coverage of access to basic health services and health service utilisation with high sub-national or regional disparities.^12^ For instance, the 2019 Ethiopian Mini-Demography and Health Survey (EMDHS) indicated that about 43% of women had received ANC4+ visits.^13^ Similarly, a study conducted in Ethiopia using Ethiopian Service Provision Assessment (ESPA) 2014 and EDHS 2016 data showed that EC of ANC services in Ethiopia was 21.5%.^14^ Another study in Ethiopia was also conducted using performance monitoring for action (PMA) data in which the EC of input and quality-adjusted ANC4+ were 28% and 12%, respectively.^15^

The Ethiopian government launched a health sector transformation plan to achieve the 2030 SDG targets.^16^ However, the existing evidence on the EC of ANC in Ethiopia often lacks a comprehensive assessment of the quality of ANC. In particular, the outcome component was overlooked and the process component only partially evaluated in the assessment of quality in the estimation of EC of ANC in Ethiopia.^15^ Another study conducted in Ethiopia did not consider the experience of care or satisfaction and structural component in the quality of ANC measurement.^14^ This limited assessment of quality of ANC can either overestimate or underestimate both the quality of ANC and the EC of ANC4+ services. To address these gaps, our study considered the three domains of the Donabedian quality care model: structure (readiness), process (adherence to standard care), and outcome (satisfaction or experience of care).^17^ This approach provides a more comprehensive evaluation of ANC quality that in turn helps to estimate EC. Therefore, this study aimed to measure the EC of ANC4+ using EMDHS 2019 and ESPA 2021–2022 data.

## Materials and methods

### Study design and settings

We conducted a secondary data analysis of the cross-sectional surveys using data from the 2019 EMDHS and the 2021–2022 ESPA. This study was reported adhering to the STROBE checklist for cross-sectional studies (Supplemental Table 1).^18^ The study was conducted across Ethiopia, including Afar, Amhara, Benishangul-Gumuz, Gambela, Harari, Oromia, Somali, former Southern Nations, Nationalities, and Peoples that includes the current Sidama, Central Ethiopia, South Ethiopia, and South-West Ethiopia regions and two city administrations (Addis Ababa and Dire Dawa). The healthcare system of Ethiopia is organised into a three-tier system: level 1 (district health system or primary healthcare unit) is comprised of a primary hospital and health centres with their satellite health posts. Level 2 is composed of general hospitals, and level 3 is made up of specialised hospitals comprised of federally run, specialised hospitals, and university hospitals.

### Sample and data sources

In this study, all women aged 15–49 year for whom their most recent birth was a live birth during the EMDHS 2019 survey period were eligible for inclusion in the study cohort to estimate the CC of ANC4+. Pregnant women who attended ANC at healthcare facilities included in ESPA 2021–2022 were eligible for inclusion in the cohort used to calculate the quality of care. EC of ANC4+ was the outcome variable of this study. EMDHS 2019 and ESPA 2021–2022 data were used to measure the EC of ANC4+. Both the EMDHS 2019 and ESPA 2021–2022 data are publicly available at https://www.dhsprogram.com/data/dataset_admin/login_main.cfm. The 2019 EMDHS is a nationally representative household survey data conducted in Ethiopia that focused on the collection of information on key performance monitoring indicators.

In EDHS data collection, enumeration areas (EAs) were used as a sampling frame. EA is a geographical unit that covers an average of 131 households in DHS of Ethiopia. In the EMDHS 2019, each region was stratified into urban and rural areas, resulting in a total of 21 sampling strata. A total of 305 EAs (93 urban and 212 rural) were selected independently in each stratum in two stages proportional to the stratum’s size. Appropriate variables were extracted from the Kid’s Record file of EMDHS 2019 dataset. Ultimately, a total weighted sample of 3617 women aged 15–19 years whose most recent birth was a live birth as recorded in EMDHS 2019 were eligible for the analysis. The details of the sampling procedures are described in the EMDHS 2019 report.^13^

The ESPA 2021–2022 survey is a national representative facility-based survey that collects various key performance indicators on the service provision at health facilities. The 2021–2022 ESPA is designed to provide representative results for each of Ethiopia’s regions separately, for all facilities together and by facility type at the national level, that is, hospitals (both public and private hospitals), health centres, clinics, and health posts. In the ESPA 2021–2022, a total of 1407 health facilities (413 hospitals, 310 health centres, 356 clinics, and 328 health posts) were selected using a stratified systematic random sampling technique. Stratification was achieved by first separating the health facilities in each region by facility type. All the clinics in each region were also further stratified by clinic designation (higher, medium, lower clinics, or specialty clinics). By facility type, all hospitals and higher clinics were included in the sample, because of their small number as well as the important role the hospitals played in the healthcare system. All the health centres in Dire Dawa and Harari and all clinics in Harari region were included in the sample because of their small number.

Out of 1407 selected facilities, 249 facilities were either permanently closed, not yet operational, under security issues, or had been converted into a COVID Centre. As such, data were collected only from 1158 health facilities. Regarding the health facilities that were included in our analysis, 253 did not provide ANC services, 242 did not assess either providers’ adherence to guidelines or client acceptability, and an additional 4 facilities did not assess providers’ adherence to guidelines from 1158 health facilities. Eventually, 659 health facilities in SPA 2021–2022 were included in this analysis. The details of the sampling procedures were described in the reports of ESPA 2021–2022.^19^ The samples during observation and exit interview were selected using systematic sampling based on the number of clients estimated to attend on the day of the survey. An exit interview with consumers was conducted among observed clients before they left the facility. In our analysis, only health facilities that were evaluated through the survey using structural (readiness), process, and exit interviews to assess women’s experience of care were included.

### Inclusion and exclusion criteria

In the EMDHS 2019, all women whose most recent birth during the survey period was a live birth were included in the analysis. Women who had a missing data, including “do not know” responses were excluded from the analysis. In addition, women from the Tigray region were excluded from the analysis. In ESPA 2021–2022, those health facilities that were evaluated using three quality domains – structure (readiness), process, and exit interview to assess women’s experience of care during the survey – were included in the analysis. Health facilities that evaluated only one or two of the three quality domains were excluded from the analysis.

### Statistical analysis

Stata software was used to analyse the data (Stata Now 19 SE-Standard Edition; Stata Corp LLC, College Station, TX, USA). Moreover, ArcGIS Pro 3.4 (Esri) software was used to display EC of ANC4+ across administrative regions of Ethiopia. After data cleaning of ESPA 2021–2022 data, the aggregated quality of ANC (combination of readiness, process, and experience of care) and each quality domain of ANC were weighted using facility weight. In addition, the process and experience of care domains of quality of ANC were weighted using observation and exit-interview weights of ESPA 2021–2022 to maintain representativeness, respectively.^20^ Observation and exit-interview weights were used as a sensitivity analysis to compare the findings of its corresponding facility-weighted process and experience of care domains. Importantly, facility weighting was used for the structural (readiness) component since the data for structural (readiness) domain indicators were collected at the health facility level. Observation weighting was used for the process component since the process data was collected through observation that is client level data. Exit-interview weighting was used for the experience of care (satisfaction) domain since the data was collected through exit interview which is also a client level data. Facility weighting for the process and experience of care indicators was also conducted after condensing the observation and exit-interview data into health facility level data. We combined the three quality domains after condensing the process and experience of care domains to create one quality score per health facility. Finally, we also used facility weighting for these combined quality data. The EMDHS 2019 data were weighted using individual woman’s sample weight to keep its representativeness. EC of ANC4+ was measured using CC from the EMDHS 2019 and the quality of care from the ESPA 2021–2022 data. EC was estimated using the formula

[9] EC=Q*U/N

where EC refers to effective coverage, *N* is the population in need (in this case, women who need ANC during pregnancy for their most recent live birth during the survey reference period), *U* is ANC4+ service user women for their most recent live birth during pregnancy, and *Q* refers to the quality of care, measured by the health gain achieved from the service. EC of ANC4+ was calculated using facility sample weight adjustments of the overall combined and each of structure, process, and experience of care components. Similarly, EC of ANC4+ was estimated using observation weight for process and exit-interview weight for experience of care domains as a sensitivity analysis to compare the findings for their corresponding EC of ANC4+ of facility-weighted process and experience of care.

#### Crude coverage of ANC4+

The EMDHS 2019 data was used to estimate the CC of ANC4+. In this case, CC is the proportion of women with live birth for their most recent birth who received ANC4+ service among those in need irrespective of the quality of care. CC is considered the traditional coverage and is calculated using the *U*/*N* ratio (use divided by need), which represents the proportion of women with live birth for their most recent birth who received ANC4+. The national and regional CC of ANC4+ were also determined in Ethiopia.

#### Quality of ANC

Quality of health care is the health gain from a certain healthcare service. We used WHO guidelines,^21,22^ Ethiopian National ANC guideline,^23^ and other published report^19^ to identify the key indicators (parameters) to assess the quality of ANC. We measured the quality of ANC services using data from the ESPA 2021–2022 survey, guided by the three Donabedian framework components: structure (readiness), process, and outcome (client experience of care or satisfaction).^17^ A total of 75 indicators were selected from the ESPA dataset. The structure component included 27 indicators to evaluate the readiness for ANC services. The process domain consisted of 37 indicators that assessed healthcare providers’ adherence to ANC standard guidelines. The outcome (experience of care) domain comprised 11 indicators that measured women’s experience of care (satisfaction) during their ANC visit.

A weighted additive method adapted from a DHS Methodological Report^24^ was used to compute the overall quality index of ANC. The weighted additive method considers the relative contributions of indicators that may carry out across readiness, process, and experience of care domains. We assigned weights to each quality domain based on the number of indicators and adjusted them to account for variations in the number of indicators within each domain. The quality index of each domain was then calculated by dividing the total number of all “success” or “yes” responses for the indicators within the domain by the total number of indicators and then multiplying by 100. Thus, we determined that the readiness, process, and experience of care domains were assigned weights of 36.0% (27/75 × 100), 49.3% (37/75 × 100), and 14.7% (11/75 × 100), respectively. Similarly, the quality index of each domain was determined by dividing the total number of “yes” responses for the indicators within a specific domain by the total number of indicators in that specific domain and then multiplied by its corresponding domain weight.^25^ For example, the quality index of the structure domain:

$$\mathrm{Quality}\;\mathrm{index}\;\mathrm{in}\;\mathrm{the}\;\mathrm{structure}\;(\mathrm{readiness})\;\mathrm{component}\;=\frac{\mathrm{Total}\;\mathrm{number}\;\mathrm{of}\;“\mathrm{yes}\;”\;\mathrm{responses}\;\mathrm{in}\;\mathrm{the}\;\mathrm{structure}\;\mathrm{component}}{\mathrm{Total}\;\mathrm{number}\;\mathrm{of}\;\mathrm{indicators}\;\mathrm{in}\;\mathrm{the}\;\mathrm{structure}\;\mathrm{component}}*\;36\%$$

Likewise, we applied the above formula to estimate the quality index of the rest of the process and experience of care domains, as follows:

$$\mathrm{Quality}\;\mathrm{index}\;\mathrm{in}\;\mathrm{the}\;\mathrm{process}\;\mathrm{component}=\frac{\mathrm{Total}\;\mathrm{number}\;\mathrm{of}\;“\mathrm{yes}”\;\mathrm{responses}\;\mathrm{in}\;\mathrm{the}\;\mathrm{process}\;\mathrm{component}}{\mathrm{Total}\;\mathrm{number}\;\mathrm{of}\;\mathrm{indicators}\;\mathrm{in}\;\mathrm{the}\;\mathrm{process}\;\mathrm{component}}*\;49.3\%$$

$$\begin{array}{l} \mathrm{Quality}\;\mathrm{index}\;\mathrm{in}\;\mathrm{the}\;\mathrm{experience}\;\\ \mathrm{of}\;\mathrm{care}\;\mathrm{component}\;\;\;\;\;\;\;\;\;\;\;\;\;\;\;\;\;\;\;\;\;\;\;\;\;\;\;\;\;=\frac{\mathrm{Total}\;\mathrm{number}\;\mathrm{of}\;“\mathrm{yes}”\;\mathrm{responses}\;\mathrm{in}\;\mathrm{the}\;\mathrm{experience}\;\mathrm{of}\;\mathrm{care}\;\mathrm{component}}{\mathrm{Total}\;\mathrm{number}\;\mathrm{of}\;\mathrm{indicators}\;\mathrm{in}\;\mathrm{the}\;\mathrm{experience}\;\mathrm{of}\;\mathrm{care}\;\mathrm{component}}*\;14.7\% \end{array}$$

Eventually, the three quality domains were summed to estimate the average overall quality index of ANC services across regional and national levels. The quality of care in each domain (structure, process, and experience of care) was estimated by region, urban/rural, health facility type, and managing (operating) authority of the health facilities.

### Ethical considerations

This study used publicly available DHS data and, as such, we were only required to obtain approval from the DHS Program; we did not request consent directly from the study participants. We obtained ethical approval from the Human Research Ethics Committee of Flinders University, Australia (project number 6054).

## Results

### Characteristics of the surveys

A total weighted sample of 3617 women with a live birth in their most recent birth during EMDHS 2019 included in this analysis. In ESPA 2021–2022, a total facility-weighted sample of 659 health facilities were included in the analysis. A total exit-interview-weighted sample of 4306 ANC service consumer pregnant women participated for exit interview in the assessment of their experience of care. A total observation-weighted sample of 4163 ANC service consumer pregnant women were observed during their actual ANC service uptake (Table 1).

**Table 1.** Women with live birth in their most recent birth, facilities, and ANC user women included in the surveys.

Survey types	Survey years	Sample
ESPA	2021–2022	659 health facilities 4306 ANC users participated in an exit interview 4163 ANC users observed during their service uptake
EMDHS	2019	3617 women with a live birth in their most recent birth

EMDHS: Ethiopian Mini-Demography and Health Survey; ESPA: Ethiopian Service Provision Assessment; ANC: antenatal care.

### Crude coverage of ANC4+ across women’s characteristics

The mean age of women included in this analysis was 28.87 (±0.11 standard deviation) years. In EMDHS 2019, the overall CC of ANC and ANC4+ among women aged <19 or >40 years old with live births in their most recent birth were 72.8% (95% CI: 70.5, 75.0) and 41.5% (95% CI: 38.9, 44.0), respectively. CC of ANC4+ was lowest among women aged <19 or >40 years old with live births in their most recent birth. Somali region had the lowest CC of ANC4+ with coverage of 11.3% (95% CI: 7.8, 15.9). Addis Ababa and Dire Dawa city administrations had the highest CC of ANC4+ with coverage of 82.7% (95% CI: 77.2, 87.1) and 62.1% (95% CI: 56.1, 67.8), respectively. The least CC of ANC4+ was 31.3% (95% CI: 28.2, 34.6) among uneducated women and 18.8% (95% CI: 15.2, 23.0) among the lowest wealth groups (Table 2).

**Table 2.** Crude coverage of ANC4+ across characteristics of women using the 2019 Ethiopian Mini-Demography and Health Survey, 2025 (*n* = 3617).

Characteristics	CC of ANC4+ (95% CI)	
	No (95% CI)	Yes (95% CI)
Age of women in years		
15–19	71.5 (59.7, 80.9)	28.5 (19.1, 40.3)
20–24	56.7 (50.6, 62.6)	43.3 (37.4, 49.4)
25–29	54.5 (49.7, 59.1)	45.5 (40.9, 50.3)
30–34	56.0 (50.3, 61.5)	44.0 (38.5, 49.7)
35–39	63.4 (57.0, 69.4)	36.6 (30.6, 43.0)
40–44	65.3 (55.4, 74.1)	34.7 (25.9, 44.6)
45–49	66.4 (48.6, 80.4)	33.6 (19.6, 51.4)
Place of residence		
Urban	43.3 (37.3, 49.5)	56.7 (50.5, 62.7)
Rural	63.8 (61.1, 66.4)	36.2 (33.6, 38.9)
Region		
Afar	68.8 (63.4, 73.7)	31.2 (26.3, 36.6)
Amhara	49.1 (43.9, 54.3)	50.9 (45.7, 56.1)
Oromia	59.4 (54.8, 63.8)	40.6 (36.2, 45.2)
Somali	88.7 (84.1, 92.2)	11.3 (7.8, 15.9)
B/Gumuz	43.9 (38.2, 49.6)	56.1 (50.4, 61.8)
SNNP	65.8 (60.4, 70.9)	34.2 (29.1, 39.6)
Gambela	68.2 (61.1, 74.5)	31.8 (25.5, 38.9)
Harari	60.8 (55.2, 66.2)	39.2 (33.8, 44.8)
Addis Ababa	17.3 (12.9, 22.8)	82.7 (77.2, 87.1)
Dire Dawa	37.9 (32.2, 43.9)	62.1 (56.1, 67.8)
National [Ethiopia]	58.5 (56.0, 61.1)	41.5 (38.9, 44.0)
Educational attainment		
No education	68.7 (65.4, 71.8)	31.3 (28.2, 34.6)
Primary education	54.0 (49.5, 58.4)	46.0 (41.6, 50.5)
Secondary education	29.1 (21.4, 38.2)	70.9 (61.8, 78.6)
Higher education	21.9 (11.4, 38.0)	78.1 (62.0, 88.6)
Wealth index		
Poorest	81.2 (77.0, 84.8)	18.8 (15.2, 23.0)
Poorer	63.0 (57.5, 68.1)	37.0 (31.9, 42.5)
Middle	62.0 (56.5, 67.3)	38.0 (32.7, 43.5)
Richer	52.0 (45.7, 58.2)	48.0 (41.8, 54.3)
Richest	31.1 (25.3, 37.5)	68.9 (62.5, 74.7)

B/Gumuz: Benishangul-Gumuz; CC: crude coverage; CI: confidence interval; SNNP: Southern Nations, Nationalities, and Peoples; ANC: antenatal care.

### Quality of ANC service

Overall facility-weighted quality of ANC service coverage at the national level was 52.1% (95% CI: 51.3, 52.8) with the lowest process domain of quality care at 32.0% (95% CI: 31.1, 32.9) and the experience of care component had the highest at 83.3% (95% CI: 81.6, 84.9). The national level quality of ANC using observation-weighted process and exit-interview sample weight for experience of care components were also 32.9% (95% CI: 32.6, 33.3) and 82.7% (95% CI: 82.0, 83.3), respectively. This indicates that the largest drop in the ANC cascade occurred at the process level, where only one-third of recommended interventions were delivered. Although there were some point estimate differences observed between the facility-weighted and observation-weighted for process domain and facility-weighted and exit-interview-weighted experience of care domain, they had an overlapped 95% CI. This indicated that it is possible or suitable to apply either of the sample weighing approach. The facility-weighted quality index of each indicator of ANC service was reported (Supplemental Table 2). The overall facility-weighted quality of ANC service was relatively higher among Benishangul-Gumuz region with coverage of 60.3% (95% CI: 56.9, 63.8) and the lowest was in Gambela region with coverage of 45.7% (95% CI: 42.2, 49.1). The study also showed that Harari region had better readiness for ANC service with readiness of 78.9% (95% CI: 70.9, 86.9), while Benishangul-Gumuz had relatively higher process and experience of care components of care with a corresponding coverage of 37.3% (95% CI: 31.2, 43.3) and 96.1% (95% CI: 92.9, 99.2), respectively. Harari region had the lowest experience of quality care component with a coverage of 75.4% (95% CI: 64.2, 86.5), while Gambela had the lowest readiness and process quality domains with coverage of 56.9% (95% CI: 52.0, 61.7) and 27.8% (95% CI: 23.2, 32.4), correspondingly. Regarding quality of ANC across the type of health facilities, the overall facility-weighted quality of ANC service was relatively higher among referral and general hospitals with a corresponding quality of care coverage of 55.2% (95% CI; 51.8, 58.7) and 56.8% (95% CI: 55.2, 58.5) but health posts had the lowest quality of care coverage of 46.4% (95% CI: 43.3, 49.5; Table 3).

**Table 3.** Estimation of facility-readiness, process, experience of care (satisfaction), and overall quality by its corresponding sample weights across administrative regions, urban/rural, facility types, and managing authorities of facilities in Ethiopia, 2025.

Characteristics	Readiness	Process		Experience of care		Overall quality of ANC: facility-weighted (95% CI), *n* = 659
	Facility-weighted (95% CI), *n* = 659	Facility-weighted (95% CI), *n* = 659	Observation-weighted (95% CI), *n* = 4163	Facility-weighted (95% CI), *n* = 659	Exit-interview-weighted (95% CI), *n* = 4306	
Region						
Afar	63.9 (57.6, 70.1)	33.9 (27.9, 40.0)	36.6 (33.8, 39.5)	89.0 (83.4, 94.5)	83.9 (79.7, 88.1)	52.8 (48.2, 57.4)
Amhara	81.2 (79.0, 83.4)	34.6 (32.4, 36.9)	34.9 (33.9, 35.9)	88.9 (85.7, 92.0)	89.9 (88.6, 91.1)	59.4 (57.8, 60.9)
Oromia	63.2 (61.1, 65.3)	30.0 (28.3, 31.8)	32.0 (31.4, 32.6)	85.9 (83.1, 88.7)	83.4 (82.3, 84.4)	50.2 (48.8, 51.5)
Somali	72.0 (66.7, 77.4)	32.3 (28.0, 36.5)	33.7 (31.9, 35.5)	64.3 (54.1, 74.6)	58.3 (54.2, 62.5)	51.3 (47.1, 55.5)
B/Gumuz	77.3 (72.9, 81.7)	37.3 (31.2, 43.3)	33.3 (30.6, 35.9)	96.1 (92.9, 99.2)	93.4 (91.2, 95.6)	60.3 (56.9, 63.8)
SNNP	63.6 (61.2, 66.0)	33.8 (32.0, 35.6)	33.6 (32.9, 34.3)	80.6 (77.2, 84.0)	81.4 (80.1, 82.6)	51.4 (50.0, 52.8)
Gambela	56.9 (52.0, 61.7)	27.8 (23.2, 32.4)	30.3 (27.8, 32.8)	78.4 (69.5, 86.9)	80.3 (76.4, 84.2)	45.7 (42.2, 49.1)
Harari	78.9 (70.9, 86.9)	31.7 (26.1, 37.3)	33.1 (30.3, 35.9)	75.4 (64.2, 86.5)	76.6 (72.0, 81.1)	55.1 (50.5, 59.7)
Addis Ababa	73.4 (69.6, 77.1)	33.2 (30.1, 36.2)	31.5 (30.3, 32.8)	81.5 (76.6, 86.5)	81.7 (79.8, 83.6)	54.8 (52.3, 57.2)
Dire Dawa	76.3 (71.9, 80.7)	35.7 (30.7, 40.8)	36.0 (33.7, 38.3)	83.5 (71.2, 95.7)	83.0 (77.7, 88.3)	57.3 (53.3, 61.4)
National [Ethiopia]	66.8 (65.6, 68.0)	32.0 (31.1, 32.9)	32.9 (32.6, 33.3)	83.3 (81.6, 84.9)	82.7 (82.0, 83.3)	52.1 (51.3, 52.8)
Placement of facilities						
Urban	70.9 (69.5, 72.4)	33.4 (32.2, 34.7)	32.8 (32.3, 33.2)	81.3 (79.1, 83.4)	81.4 (80.6, 82.2)	54.0 (53.0, 54.9)
Rural	65.1 (63.2, 67.0)	31.4 (30.0, 32.9)	33.1 (32.4, 33.7)	84.1 (81.5, 86.7)	83.6 (82.5, 84.7)	51.3 (50.1, 52.5)
Facility type						
Referral hospital	78.2 (73.4, 82.9)	32.8 (28.8, 36.8)	33.8 (32.5, 35.1)	74.3 (64.3, 84.3)	80.7 (78.5, 82.8)	55.2 (51.8, 58.7)
General hospital	77.8 (75.5, 80.1)	33.6 (31.3, 35.9)	34.1 (33.3, 34.9)	83.2 (79.2, 87.2)	79.6 (78.1, 81.1)	56.8 (55.2, 58.5)
Primary hospital	69.6 (67.3, 71.9)	34.3 (32.8, 35.7)	33.0 (32.4, 33.5)	82.9 (80.3, 85.4)	83.8 (82.8, 84.7)	54.1 (52.8, 55.4)
Health centre	71.6 (69.6, 73.6)	33.7 (32.2, 35.3)	33.8 (33.1, 34.5)	85.0 (82.5, 87.4)	84.2 (82.9, 85.5)	54.9 (53.7, 56.1)
Health post	57.1 (53.4, 60.8)	28.7 (24.6, 32.7)	29.9 (27.5, 32.4)	79.7 (71.4, 88.1)	76.8 (71.5, 82.2)	46.4 (43.3, 49.5)
Private clinics^a^	60.8 (57.1, 64.5)	29.5 (25.6, 32.8)	28.5 (26.6, 30.4)	85.3 (78.1, 92.41)	81.7 (78.6, 84.8)	48.8 (46.1, 51.6)
Managing authority						
Government or Public	67.2 (65.9, 68.5)	32.2 (31.1, 33.2)	33.2 (32.8, 33.6)	83.2 (81.3, 85.0)	82.5 (81.8, 83.2)	52.3 (51.4, 53.1)
Other governmental^b^	86.4 (79.2, 93.6)	35.1 (27.5, 42.8)	35.0 (31.4, 38.6)	81.8 (68.8, 94.9)	86.5 (81.1, 91.8)	60.5 (54.8, 66.1)
Private-for-profit	59.7 (57.1, 62.4)	30.8 (28.5, 33.0)	30.3 (29.2, 31.3)	83.6 (79.2, 88.1)	84.0 (82.4, 85.6)	49.0 (47.1, 50.9)
NGO^c^	79.5 (75.5, 83.4)	27.8 (21.9, 33.7)	30.5 (28.3, 32.7)	88.9 (81.1, 96.6)	84.6 (81.4, 87.9)	55.4 (51.8, 58.9)

Overall quality of ANC is the summation of the three weighted domains including readiness (36%), process or adherence (49.3%), and experience of care (14.7%). SNNP: Southern Nations, Nationalities, and Peoples; CI: confidence interval; ANC: antenatal care.

a Private clinics include specialty, higher, medium, and lower clinics.

b Other governmental organisations include Military, Prison, and Federal Police health facilities.

c NGO stands for non-governmental organisations which include mission or faith-based, and non-profit health facilities.

### Effective coverage of ANC4+ cascading

**Figure 1.**
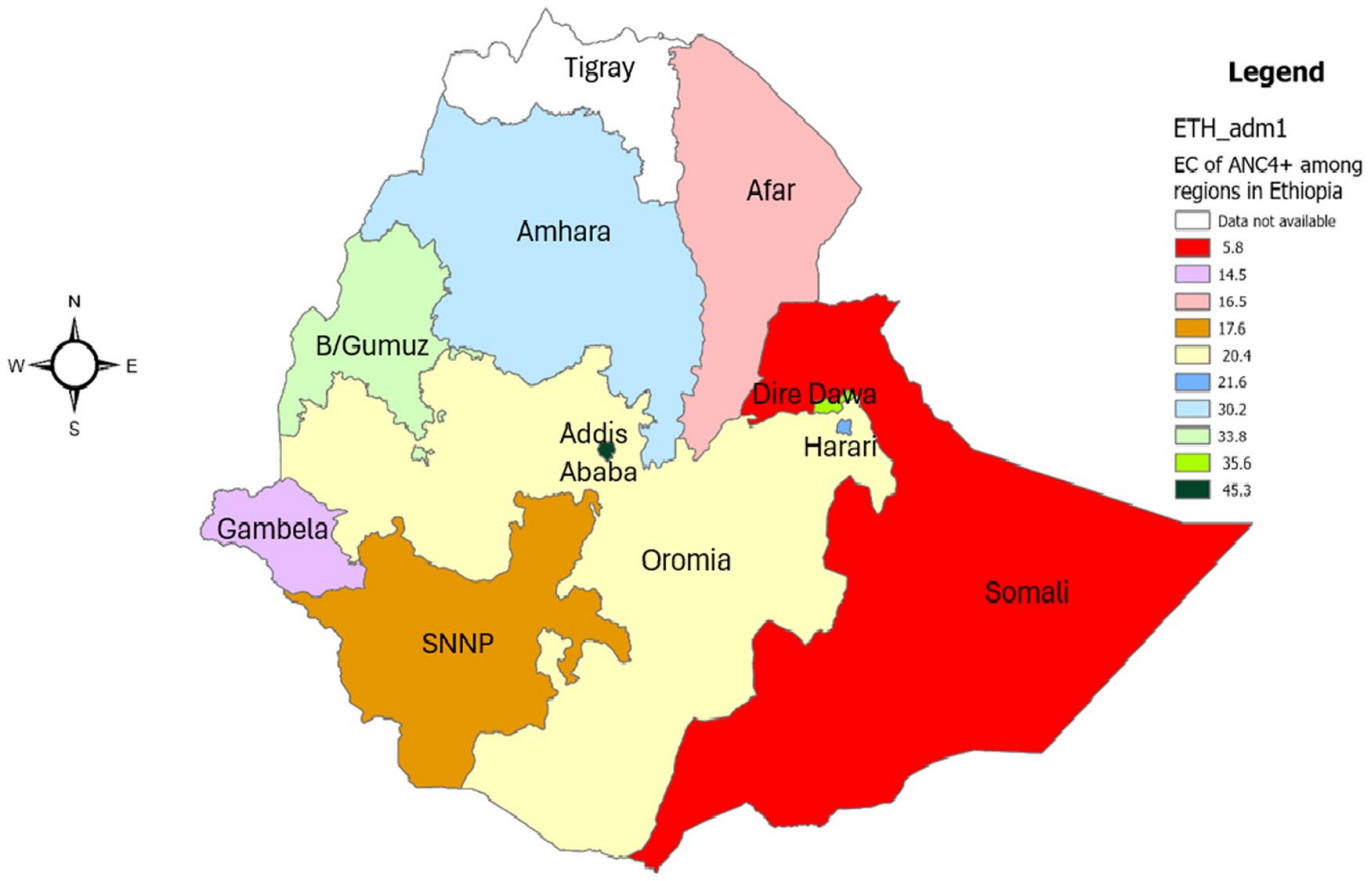
EC of four or more antenatal care (ANC4+) across the administrative regions in Ethiopia. EC: effective coverage.

**Figure 2.**
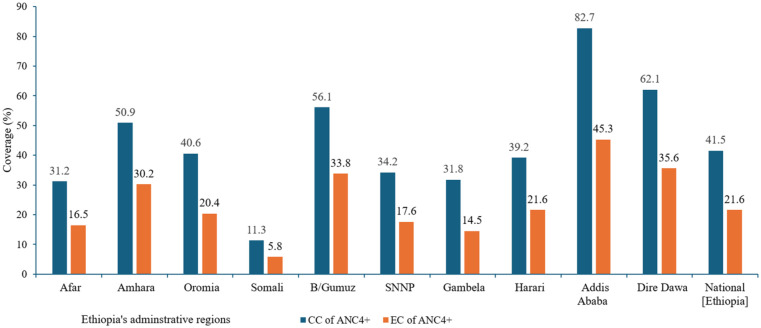
CC and EC of four or more antenatal care (ANC4+) across the administrative regions in Ethiopia. CC: crude coverage; EC: effective coverage.

**Figure 3.**
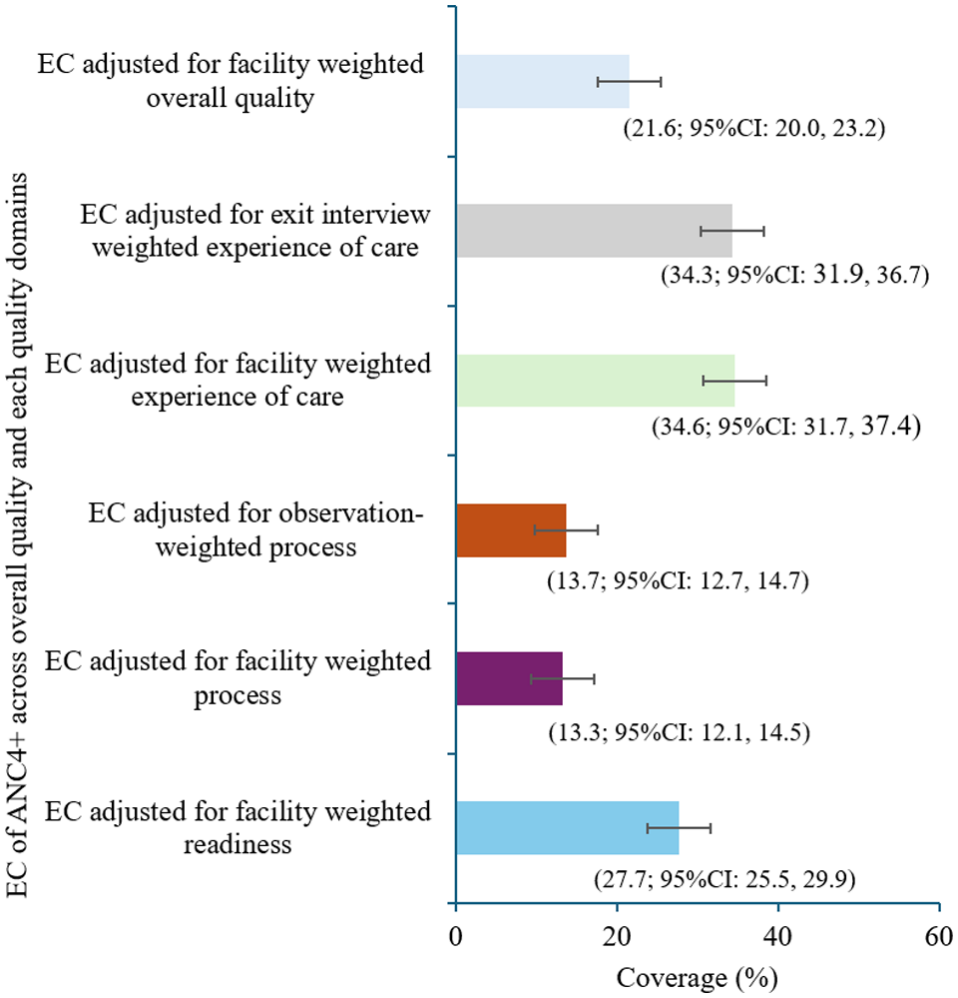
EC of four or more antenatal care (ANC4+) of Ethiopia using facility-weighted overall quality, readiness, process, and experience of care as well as observation-weighted process and exit-interview-weighted experience of care. EC: effective coverage.

The overall national level EC of ANC4+ was 21.6% (95% CI: 20.0, 23.2) with the lowest EC of 5.8% (95% CI: 3.7, 8.8) in Somali region and the highest EC of 45.3% (95% CI: 40.4, 49.8) in Addis Ababa city administration (Table 4). EC of ANC4+ was depicted using administrative region map of Ethiopia (Figure 1). The gap between CC (41.5%) and EC (21.6%; Figure 2) shows that nearly half of women attending ANC4+ visits did not receive quality-adjusted care. This finding suggests that nearly half of the women who achieved ANC4+ contacts did not qualify the care when the quality of ANC was taken into consideration. The low EC of ANC4+ in Somali may be associated with many rural communities in the region being pastoralists, while Ethiopia’s healthcare system is not designed to provide sufficient services to mobile pastoral populations. This limits their access to essential services, including ANC which leads to structural and quality care inequalities. The EC of ANC4+ using facility-weighted adjustments across each quality domains was 27.7% (95% CI: 25.5, 29.9) for readiness, 13.3% (95% CI: 12.1, 14.5) for process, and 34.6% (95% CI: 31.7, 37.4) for experience of care. Similarly, the EC of ANC4+ based on observation-weight adjustments was 13.7% (95% CI: 12.7, 14.7) for the process component, and the estimate based on exit-interview weights was 34.3% (95% CI: 31.9, 36.7) for experience of care (Figure 3).

**Table 4.** Cascading EC of ANC4+ using facility-weighted quality of ANC from ESPA 2021–2022 and CC of ANC4+ from EMDHS 2019 data in Ethiopia, 2025.

Characteristics	Overall facility-weighted quality of ANC (95% CI)	Overall facility-weighted quality index (95% CI)	CC of ANC4+ (95% CI)	EC of ANC4+ (95% CI)
Region				
Afar	52.8 (48.2, 57.4)	0.528 (0.482, 0.574)	31.2 (26.3, 36.6)	16.5 (12.7, 21.0)
Amhara	59.4 (57.8, 60.9)	0.594 (0.578, 0.609)	50.9 (45.7, 56.1)	30.2 (26.4, 34.2)
Oromia	50.2 (48.8, 51.5)	0.502 (0.488, 0.515)	40.6 (36.2, 45.2)	20.4 (17.7, 23.3)
Somali	51.3 (47.1, 55.5)	0.513 (0.471, 0.555)	11.3 (7.8, 15.9)	5.8 (3.7, 8.8)
B/Gumuz	60.3 (56.9, 63.8)	0.603 (0.569, 0.638)	56.1 (50.4, 61.8)	33.8 (28.7, 39.4)
SNNP	51.4 (50.0, 52.8)	0.514 (0.50, 0.528)	34.2 (29.1, 39.6)	17.6 (14.6, 20.9)
Gambela	45.7 (42.2, 49.1)	0.457 (0.422, 0.491)	31.8 (25.5, 38.9)	14.5 (10.8, 19.1)
Harari	55.1 (50.5, 59.7)	0.551 (0.505, 0.597)	39.2 (33.8, 44.8)	21.6 (17.1, 26.7)
Addis Ababa	54.8 (52.3, 57.2)	0.548 (0.523, 0.572)	82.7 (77.2, 87.1)	45.3 (40.4, 49.8)
Dire Dawa	57.3 (53.3, 61.4)	0.573 (0.533, 0.614)	62.1 (56.1, 67.8)	35.6 (29.9, 41.6)
National [Ethiopia]	52.1 (51.3, 52.8)	0.521 (0.513, 0.528)	41.5 (38.9, 44.0)	21.6 (20.0, 23.2)

CC: crude coverage; EC: effective coverage; EMDHS: Ethiopian Mini-Demography and Health Survey; ESPA: Ethiopian Service Provision Assessment; SNNP: Southern Nations, Nationalities, and Peoples; CI: confidence interval; ANC: antenatal care.

## Discussion

Reducing maternal morbidity and mortality requires a health system that delivers not only wide coverage but also high-quality care. Therefore, this study assessed the EC of ANC4+ in Ethiopia using nationally representative data of ESPA 2021–2022 and EMDHS 2019. CC estimates do not put into account service quality and therefore fail to indicate the potential health gain of women from attending ANC. EC reflects the link between service use and the quality of care provided. As such, the overall CC of ANC4+ was 41.5%, but only 21.6% received EC of ANC4+, indicating that 50% of women did not receive quality care. Our study also showed that the overall facility-weighted average quality of ANC service at the national level was 52.1%. EC of ANC4+ adjusted for facility-weighted readiness, process, and experience of care for ANC were 27.7%, 13.3%, and 34.6%, respectively. The EC of ANC4+ indicated substantial disparities across regions with the lowest in Somali region and the highest coverage observed in Addis Ababa.

EC is one of the measures of UHC, as it accounts for both service utilisation and quality of care. In this study, only 21.6% (95% CI: 20.0, 23.2) of women with their most recent live birth received EC of ANC4+, which was far below the 80% UHC essential health service coverage target set for 2030.^8,26^ This means that ANC4+ contact without quality is insufficient to prevent maternal morbidity and mortality. Our finding also highlights a large gap between CC and EC of ANC4+ with the greatest loss occurred at process component. This suggests that health system strengthening in Ethiopia should prioritise provider adherence to ANC protocols rather than only focusing on expanding coverage. This magnitude of this finding is consistent with the EC of ANC conducted using ESPA 2014 and EDHS 2016 in Ethiopia (21.5%).^14^ However, this finding is higher than a study conducted using the PMA data in Ethiopia with EC of ANC4+ of 12%^15^ and lower than the EC of ANC4+ in Kenya in 2014 (44.6%), 2008–2009 (31.6%), and 2003 (31.7%),^27^ EC of ANC in Uganda (38%),^28^ and Ethiopia’s UHC reproductive, maternal, neonatal, and child health service coverage (37.5%).^29^ These low EC may be partly explained by poor adherence to ANC guideline that limits the women from getting potential health gain.^30^ The difference across studies could also be attributed to the variations in study settings, study period, research design, and participant characteristics. Another justification for the variations in EC of ANC may be due to the differences in measurement of quality, facility-readiness, or financing systems of maternity care.

The national level CC of ANC was 72.8% (95% CI: 70.5, 75.0). The proportion of women in Ethiopia who received first ANC visit from a skilled provider has increased from 27% in 2000,^31^ 28% in 2005,^32^ 34% in 2011,^33^ and 62% in 2016.^5^ As shown in the previous EDHS reports, the current finding showed significant improvement in the coverage of first ANC visit despite only 41.5% (95% CI: 38.9, 44.0) of Ethiopian women completed the recommended four or more ANC visits. This finding is in line with a study conducted in Ethiopia using PMA data with CC of ANC4+ (40%)^15^, however, lower than a study finding in SSA (54.5%).^34^ This low CC of ANC4+ was likely due to the political instability and conflict disrupting access to ANC. The possible reasons for such low completion of the recommended ANC visits might also be attributed to inadequate healthcare infrastructure, poor quality of care, long distance to health facility, and limited transportation access.^35–37^

The overall quality coverage of ANC in Ethiopia is low despite it varies across the administrative regions of Ethiopia. The overall facility-weighted quality of ANC was 52.1% (95% CI: 51.3, 52.8), which was higher than the studies conducted in Ethiopia using ESPA 2014 data (34.4%)^14^ Ethiopia using EDHS 2016 data (22.5%),^38^ Wogera district Ethiopia (32.7%),^39^ East Africa (11.2%),^40^ and Nepal (43%).^41^ In contrast, our study finding was lower than a study conducted in northwest Ethiopia (76.7%).^42^ In our study, facility-weighted process quality domain was the lowest ANC quality component at 32.0% (95% CI: 31.1, 32.9). This was in line with a study conducted on adherence to ANC service in Gondar town, Ethiopia (32.3%)^43^ and lower than a study conducted in northwest Ethiopia (77.8%).^42^ This low adherence to standard quality of ANC indicates that Ethiopian women likely obtain suboptimal clinical actions and interactions during ANC visits, including insufficient self-care guidance, inadequate communication between providers and women, and gaps in check-ups, counselling, and screenings. Lack of adherence to ANC guideline reduces the potential health benefits for women who utilise ANC service.^21^ The quality of ANC services varied across types of health facilities with relatively higher among hospitals. Health posts had also lower ANC service quality (46.4%). The observed variation in the quality of ANC services across health facility types can be attributed to differences in resource availability, infrastructure, staffs, and provider competency. Hospitals are generally better equipped with diagnostic tools, skilled healthcare personnel, and clinical guidelines, which enable them to provide more comprehensive and standardised ANC services.^22^

In this study, EC of ANC4+ service utilisation showed marked disparities across administrative regions in Ethiopia. The highest EC of ANC4+ was observed in Addis Ababa and Dire Dawa city administrations. These findings may be attributed to higher health workforce density, better infrastructure, better health literacy, and improved health-seeking behaviours commonly higher in urban settings.^44^ Conversely, the lowest EC of ANC4+ were reported in the Somali and Gambela regions. The possible reasons for such low coverage could be related with lack of awareness, weak social support, wrong beliefs, long distance, poor access to transportation, poor approaches of caregivers, long waiting time at health facilities, and inadequate skill of providers.^45^

### Limitations

This article adds a methodological value on the EC estimation by measuring the quality of ANC service using the three components of Donabedian quality domains at the national level. However, the study has some limitations. The study did not account for the changes associated with the time gap between the EMDHS 2019 and the ESPA 2021–2022 surveys. As such, the quality of ANC service delivery in 2021–2022 might be different before 2019 due to COVID-19 pandemic and service disruptions due to ongoing conflicts in Ethiopia. Using satisfaction as a proxy measure for the outcome component of quality of ANC is weak and might affect the quality estimation. Equal weighting of indicators (based on number of indicators) is also arbitrary that might mask the effect of key indicators. Exclusion of closed or insecure facilities during data collection may bias the quality estimation. Furthermore, the Tigray region might not be included in the analysis, as the ESPA 2021–2022 data were not collected in the region due to the ongoing conflict. Missing data was another limitation of the study.

## Conclusion

In this study, low EC of ANC4+ was reported linked with low proportion of women attending ANC4+ and low quality of ANC provision with high regional disparities. This can have a negative impact on Ethiopia’s progress towards UHC goal and maternal mortality and morbidity reduction. As such, Ethiopia is unlikely to meet SDG-3 targets for maternal health without urgent action. Therefore, strengthening healthcare infrastructure, increasing community engagement, and adherence to standard ANC guideline are essential to enhancing the quality of care. Employing region-specific health system strengthening interventions including provider training, quality improvement, equity in access, and ensuring accountability supported by ongoing monitoring and evaluation are also crucial to improve EC of ANC4+ in Ethiopia.

## Supplemental Material

sj-docx-1-whe-10.1177_17455057261438733 – Supplemental material for Effective coverage of antenatal care service utilisation in Ethiopia using service provision assessment and demography and health survey data Supplemental material, sj-docx-1-whe-10.1177_17455057261438733 for Effective coverage of antenatal care service utilisation in Ethiopia using service provision assessment and demography and health survey data by Ayal Debie, Molla M. Wassie, Claire T. Roberts, Annabelle Wilson and Jacqueline H. Stephens in Women's Health

sj-docx-2-whe-10.1177_17455057261438733 – Supplemental material for Effective coverage of antenatal care service utilisation in Ethiopia using service provision assessment and demography and health survey data Supplemental material, sj-docx-2-whe-10.1177_17455057261438733 for Effective coverage of antenatal care service utilisation in Ethiopia using service provision assessment and demography and health survey data by Ayal Debie, Molla M. Wassie, Claire T. Roberts, Annabelle Wilson and Jacqueline H. Stephens in Women's Health
