# Supplementary material for: Effective coverage of antenatal care service utilisation in Ethiopia using service provision assessment and demography and health survey data
Source: Womens Health (Lond). 2026 Jul 24;22:17455057261438733. doi: 10.1177/17455057261438733 (PMC13400925; doi:10.1177/17455057261438733)
Supplement: sj-docx-2-whe-10.1177_17455057261438733 – Supplemental material for Effective coverage of antenatal care service utilisation in Ethiopia using service provision assessment and demography and health survey data [file sj-docx-2-whe-10.1177_17455057261438733.docx]

Supplementary Table 2: ANC service quality score using ESPA 2021-22 data in Ethiopia

| Domain | Quality assessment indicators | Facility weighted-quality score |
| --- | --- | --- |
| Readiness  (Structure) | Visual aid for health education | 0.581 |
|  | Tape measure for fundal | 0.683 |
|  | ANC national guideline | 0.512 |
|  | Examination bed | 0.917 |
|  | Examination light | 0.545 |
|  | Adult weight scale | 0.874 |
|  | Fetal stethoscope | 0.898 |
|  | Stethoscope | 0.849 |
|  | BP apparatus (Digital or Manual) | 0.792 |
|  | Integrated Treated Bed Nets/ Long-Lasting Insecticidal Nets | 0.242 |
|  | Tetanus Toxoid | 0.807 |
|  | Iron or Folic acid or combined | 0.785 |
|  | Standard Precaution Infection Prevention Guideline | 0.377 |
|  | Health provider at facility 24 hours per day | 0.741 |
|  | Provider received ANC training | 0.257 |
|  | Eye protection | 0.276 |
|  | Gowns | 0.878 |
|  | Medical Masks | 0.781 |
|  | Alcohol handrub | 0.837 |
|  | Pedal bin waste | 0.580 |
|  | Auto disable (AD) syringes | 0.860 |
|  | Disinfectant | 0.779 |
|  | Gloves | 0.887 |
|  | Sharps container | 0.934 |
|  | Soap | 0.452 |
|  | Running water | 0.407 |
|  | Electric power | 0.619 |
| Process | Asked age | 0.381 |
|  | Asked current medications | 0.202 |
|  | Asked date of last menstrual period | 0.502 |
|  | Asked number of prior pregnancies | 0.423 |
|  | Measured Blood Pressure | 0.669 |
|  | Checked fetal presentation | 0.540 |
|  | Palpated fundal height | 0.665 |
|  | Checked fetal heartbeat | 0.642 |
|  | Measured weight of the client | 0.765 |
|  | Examined conjunctiva | 0.305 |
|  | Examined edema | 0.186 |
|  | Examined breasts | 0.048 |
|  | Conduct perineal care or vaginal examination | 0.008 |
|  | Examined swollen glands | 0.057 |
|  | Conducted or referred ultrasound | 0.118 |
|  | Provider discussed about food eaten during pregnancy | 0.519 |
|  | Provider informed the client about progress of pregnancy | 0.376 |
|  | The provider discussed the importance of at least 4 ANC visits | 0.171 |
|  | Measured fundal height | 0.081 |
|  | Washed hands with soap or use alcohol hand rub | 0.162 |
|  | Explained the procedure to perform | 0.178 |
|  | Explained why the procedure was needed | 0.075 |
|  | Obtained permission before procedure | 0.118 |
|  | Prescribed either iron or folic or combined | 0.612 |
|  | Prescribed tetanus toxoid | 0.454 |
|  | Prescribed Mebendazole | 0.036 |
|  | Provider advised or counselled about skilled delivery | 0.146 |
|  | Wrote on client health card | 0.866 |
|  | Performed or referred Hct/Hgb test | 0.334 |
|  | Performed or referred urine test | 0.305 |
|  | Performed or referred for VDRL test | 0.008 |
|  | Performed or referred for blood group test | 0.355 |
|  | Asked about HIV status | 0.233 |
|  | Performed or referred for HIV test | 0.302 |
|  | Provide ITN to client or instructed the client to obtain ITN | 0.020 |
|  | Asked about at least one of prior pregnancy complications | 0.402 |
|  | Asked at least one of the current pregnancy complications | 0.636 |
| Experience of care (Outcome) | Satisfied with timely care | 0.751 |
|  | Satisfied with the ability to discuss problems or concerns | 0.797 |
|  | Satisfied with the amount of explanation for the problem or treatment | 0.821 |
|  | Satisfied with the visual privacy | 0.900 |
|  | Satisfied with the auditory privacy | 0.852 |
|  | Satisfied with the availability of medicine | 0.780 |
|  | Satisfied with working hours | 0.785 |
|  | Satisfied on working days | 0.806 |
|  | Satisfied with cleanliness of health facilities | 0.789 |
|  | Satisfied with treatment of clients | 0.904 |
|  | Satisfied with cost of services | 0.978 |
